# Comparison of the effect of oral pregabalin with intravenous ketamine on reducing acute pain after abdominal hysterectomy: A randomized double-blind clinical trial

**DOI:** 10.22088/cjim.12.2.217

**Published:** 2021-03

**Authors:** Negin Ghadami, Farzad Sarshivi, Arvin Barzanji, Bijan Nouri, Zahra Mohammadi

**Affiliations:** 1 Department of Anesthesiology, Faculty of Medicine, Kurdistan University of Medical Sciences, Sanandaj, Iran; 2 Social Determinants of Health Research Center, Kurdistan University of Medical Sciences, Sanandaj, Iran

**Keywords:** Pregabalin, Ketamine, Pain, Hysterectomy

## Abstract

**Background:**

The aim of this study was to compare the analgesic effects of pregabalin and ketamine on reducing pain after abdominal hysterectomy.

**Methods:**

In this double-blind clinical trial, one hundred forty ASA I and II patients of age range 30-60 years scheduled for abdominal hysterectomy undergoing general anesthesia in 2018, were randomly divided into 4 equal groups. Pregabalin group received 300 mg oral pregabalin, ketamine group received 0.3 mg/kg of intravenous ketamine, and pregabalin- ketamine group received the combination of the two-above medication, and placebo group received the placebo and saline. Patients were evaluated for pain intensity according to the visual analogue scale (VAS) at 2, 4, 6, 12, 18, and 24 hours after surgery. Also, the need for analgesic drugs and the frequency of repetitions were also recorded. Statistical analysis was performed using STATA, Version 14. A p- value less than 0.05 was considered statistically significant.

**Results:**

In the pregabalin and pregabalin-ketamine groups, pain in the first 6 hours after the end of operation was significantly less than the other two groups (p<0.05), but there was no significant difference between the 4 groups at 18 and 24 hours after surgery. The need for analgesic medications in the pregabalin group was lower than in other groups (p<0.05).

**Conclusion:**

The results of this study show that the administration of oral pregabalin with and without intravenous ketamine before abdominal hysterectomy can decrease postoperative pain and reduce the need for analgesia.

Acute postoperative pain is one of the most common serious postoperative problems and is considered as a stressful condition for patients (1). Acute postoperative pain induces sympathetic system, increases catecholamines and stress hormones, increases blood pressure and heart rate, increases catabolism, immunosuppression, delays postoperative recovery, prolonged hospitalization, restlessness due to inability to communicate, chronic and prolonged pain, reduces the quality of postoperative life, and ultimately increases the cost of treatment for the patient and the community (2-4). Recent studies have shown to control postoperative acute pain, various medications such as gabapentinoids like as gabapentin, pregabalin, and also ketamine have been used (5-8). Pregabalin is a structural derivative of gamma-aminobutyric acid (GABA), which has analgesic, anticonvulsant, and anti-anxiety effects. Nowadays its use is in the neuropathic pain therapy (9-11).

Its mechanism of action is to connect to the pre-synaptic subgroups of alpha 2 GABA channels dependent on calcium voltage that is extensively present in the spinal cord and brain. The stimulation of the pre-synaptic groups of alpha 2 GABA prevents the release of stimulatory neurotransmitters including glutamate, norepinephrine and substance P, and thus returns the neurons that are excessively stimulated in the central nervous system to their normal state (5, 12-14)

Ketamine is a drug used for anesthesia, sedation, and analgesia. Ketamine, with an antagonistic effect on NMDA receptor (N-Methyl-D-aspartate), prevents or reverse central nervous system (CNS) sensitivity to painful stimuli and reduces postoperative pain. This drug blocks the NMDA receptor in the post-synaptic membrane of the spinal cord posterior horn and prevents pain transfer through pain fibers to the central nervous system, therefore reduces pain(15). Ketamine applies its analgesic effect by inhibiting sodium and potassium channels of peripheral nerves system(16). In several studies, the analgesic properties of ketamine for the control of perioperative acute pain have been investigated (1, 6, 16-18). Both pregabalin and ketamine effect on the glutamate pathway, but pregabalin is effective on p receptor(11). The aim of this clinical trial was to evaluate the effect of intravenous ketamine and oral pregabalin on acute postoperative pain control in patients undergoing abdominal hysterectomy surgery.

## Methods

This double-blind clinical trial was conducted at the Women's Surgery Center of Besaat Hospital, Sanandaj, Iran after obtaining the consent of the Ethics Committee of the Kurdistan University of Medical Sciences (IR.MUK.REC.1396) and was registered in the Iranian Registry of Clinical Trials (IRCT: **IRCT20180428039448N1**) in 2018. One hundred forty patients, with inclusion criteria of age range 30-60 years and American Society of Anesthesiologists (ASA) physical status I and II were selected to this study. Patients with general anesthetic contraindications, duration of surgery for more than 150 minutes, history of drug abuse, history of chronic pain and use of analgesic, antidepressants, and sedative drugs, history of drug allergy, seizure, uncontrolled blood pressure, contraindication for acetaminophen and non-steroid anti-inflammatory drugs and previous abdominal surgery were excluded from the study. Informed consent was taken from all the patients.

In this study, 140 patients were selected considering the significance level of 95%, test power of 90% and effect size of 0.8, sample size was calculated using the following equation, which is equal to 35 patients in each group. According to the following formula and referred for abdominal hysterectomy were randomly divided into 4 equal groups by the generation of random numbers done by computer and entered into the study after obtaining informed consent. This study is parallel and the allocation ratio is 1.

n=2Z1-α2+Z1-β2d2+Z1-α224

Following the selection of patients, an explanation was given on how to collaborate in the study and on the visual analog scale (VAS). The patients were randomly allocated into four groups using a block randomization method.

All patients in each group received an oral capsule with 20 ml water 90 minutes before surgery. The capsule contains 300 mg of pregabalin in pregabalin group (P) and pregabalin-ketamine (PK) groups and placebo(C) in two other groups which were custom-made in the form of pregabalin capsule filled with sugar. Also, all patients received intravenous 0.3 mg/kg of ketamine (K and PK groups) or normal saline (P and C groups) 30 minutes before surgery, of which its volume reached to 5 ml in all patients.

Patients were unaware of their grouping. Medications were provided by the anesthesiologist and administered by an anesthetist. Both of these individuals did not play a role in collecting information and evaluating patients. Data were collected by an anesthesia resident who was not aware of the patient grouping. After administration of the medications in the study groups, patients were monitored closely. Patients were also awarded on possible complications. Blood pressure and heart rate of patients were controlled at intervals of 30 minutes till 90 minutes before surgery. In the operating room, patients underwent continuous monitoring of noninvasive blood pressure, pulse oximetry, electrocardiography, and scenography. 

Before anesthesia induction, 500 ml of Ringer serum was infused to all patients. The induction of anesthesia in all patients was performed with fentanyl (2 μg/kg), propofol (2 mg/kg), A atracurium (0.5 mg/ kg), midazolam (2 mg), and lidocaine (1 mg/kg body weight). After intubation, anesthesia was maintained in both groups using 4 L/min oxygen and 1.2% isoflurane. At the end of the surgery, and reverse muscle relaxant drugs, and after the extubation, patients were transferred to recovery. For postoperative analgesia, 100 mg diclofenac suppository was used every 8 h. Also, if there was a pain more than 3, based on the VAS scale, 30 minutes after suppository placement, IV acetaminophen (1 g per 100 ml normal saline) was used, and in case of continued pain, 2.5 mg morphine was administered intravenously.

As the primary outcome, the pain was evaluated using VAS at 2, 4, 12, 6, 18, and 24 hours after the end of surgery. Also, as the secondary outcomes, the need for analgesia drug and the frequency of repetitive medications were also recorded. Changes in hemodynamic symptoms (heart rate and systolic blood pressure) were also recorded. The incidence of postoperative nausea and vomiting was also recorded.

To analyze the data, STATA software Version 14 was used. First, the concentration and frequency indices and the frequency distribution table were drawn for the quantitative and qualitative variables, respectively and then non-parametric Kruskal-Wallis test and Mann-Whitney post hoc test were used to compare the ordinal variables in the experimental groups. Furthermore, for each group, the trend of pain over time was analyzed using Friedman Test. Chi-square test was used to compare qualitative variables in experimental groups. A p-value less than 0.05 was considered statistically significant.

## Results

This study was a double-blind, clinical trial on one hundred forty patients undergoing abdominal hysterectomy surgery. Samples were randomly allocated into four groups. There were no significant differences between the four groups in terms of age, and duration of surgery (table 1).

**Figure 1:**
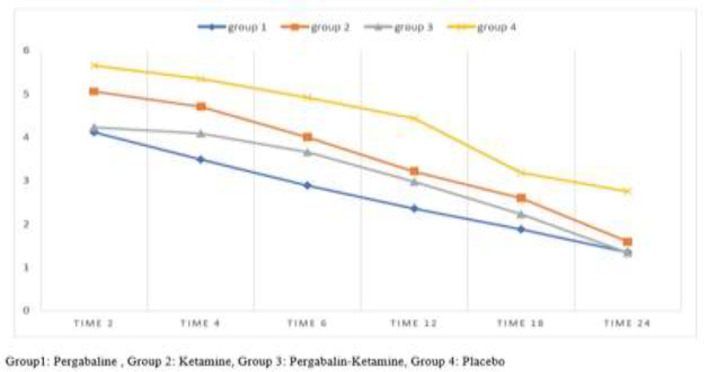
Mean pain scores changes during the times of study

Table 2 shows the comparisons of the mean (±SD) pain score in the times of study and between groups in the same time. The analytical results of the study showed that there is a significant difference between mean pain score in different times in the studied groups (figure 1). Moreover, the pain levels were compared in four groups. Based on Kruskal-Wallis test and Mann-Whitney post hoc test, there was a significant difference between the pain levels of the four groups at the same time, hence, the pain level in the pregabalin group was lower than the other groups. Besides, the pain score in study hours between the pregabalin group and ketamine group in the first 6 hours was significantly different, but no significant difference was observed at 18 and 24 hours after surgery. The mean score in the pregabalin group (P) was lower. In terms of pain score, there was no significant difference between the pregabalin group (P) and pregabalin-ketamine group (PK) in any study time, however, the mean pain score in the pregabalin group was lower. 

**Table 1:** Comparisons of the parameters of age, and duration of surgery, between the four groups of patients under abdominal hysterectomy

**P-value**	**Placebo Group**	**Pregabalin-Ketamine Group**	**Ketamine Group**	**Pregabalin Group**	**Parameter**
0.406	38.65±11.69	41.37±10.47	37.88±9.34	40.94±9.08	Age
0.734	124±15.08	125±16.92	122±15.87	127±16.05	Duration of Surgery

**Table 2:** Comparisons of the mean (±SD) pain score in times of study and between four groups of study in patients under abdominal hysterectomy

**Group**	**VAS 2h**	**VAS 4h**	**VAS 6h**	**VAS 12h**	**VAS 18h**	**VAS 24h**	**Friedman Test**
Pregabalin Group (1)	4.11 (2.59)	3.48 (2.40)	2.88 (2.34)	2.35 (1.80)	1.87 (1.43)	1.34 (1.23)	P=0.000
Ketamine Group (2)	5.05 (2.58)	4.70 (2.71)	4.00 (2.90)	3.20 (2.55)	2.59 (2.02)	1.59 (1.05)	P=0.000
Pregabalin-Ketamine Group(3)	4.22 (1.83)	4.08 (1.85)	3.65 (1.74)	2.97 (1.48)	2.22 (0.88)	1.33 (0.73)	P=0.000
Placebo Group(4)	5.65 (2.56)	5.34 (2.94)	4.91 (2.52)	4.43 (2.46)	3.18 (2.10)	2.75 (1.77)	P=0.000
Kruskal- Wallis	P=0.003	P=0.004	P=0.003	P=0.004	P=0.110	P=0.019	
Mann-Whitney	1&2, P=0.031 1&3, P=0.815 1&4, P=0.006 2&3, P=0.024 2&4, P=0.207 3&4, P=0.002	1&2, P=0.013 1&3, P=0.378 1&4, P=0.002 2&3, P=0.079 2&4, P=0.323 3&4, P=0.008	1&2, P=0.044 1&3, P=0.192 1&4, P=0.000 2&3, P=0.268 2&4, P=0.213 3&4, P=0.003	1&2, P=0.129 1&3, P=0.171 1&4, P=0.001 2&3, P=0.447 2&4, P=0.074 3&4, P=0.003		1&2, P=0.328 1&3, P=0.719 1&4, P=0.007 2&3, P=0.281 2&4, P=0.053 3&4, P=0.005	

Kruskal-Wallis test and Mann-Whitney post hoc and Friedman Test (P< 0.05)

Table 3 shows the need for additional analgesia drugs. According to the findings of this table, the lowest amount of additional analgesia received in the step two was for pregabalin group and then the pregabalin-ketamine group (PK). The incidence of nausea and vomiting in the pregabalin, ketamine, pregabalin-ketamine, and placebo groups were 8, 9, 11, and 14 people, respectively. 

**Figure 2:**
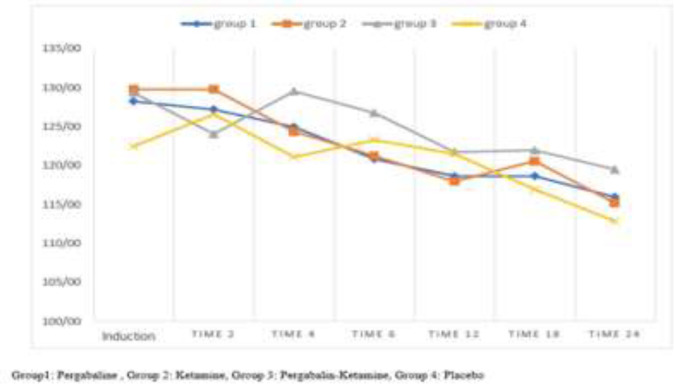
Mean changes in systolic blood pressure in different times in groups of study

The highest incidence of nausea and vomiting was in the placebo group (40%). The analytical results of the changes in mean(±SD) systolic blood pressure at different hours in pregabalin group showed that there was a significant difference between times of study. The systolic blood pressure decreased from recovery to 24 hours after surgery (p<0.001), but in the pregabalin-ketamine group, these changes were not significant (P=0.154) (figure 2).

**Figure 3:**
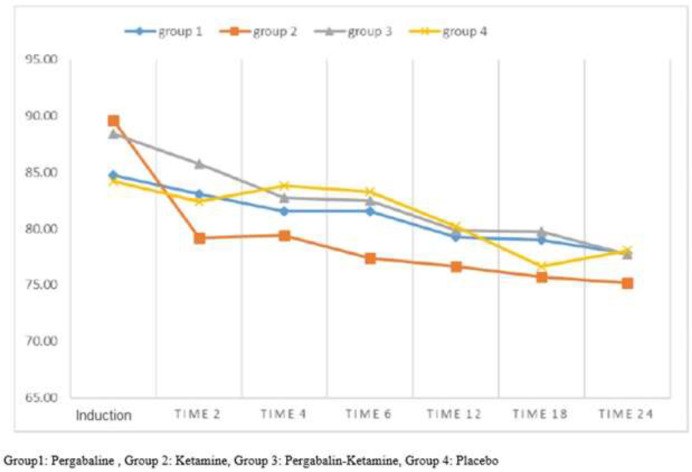
Mean changes in heart rate in different times in groups of study

Further, the analytical results from the analysis of changes in mean (±SD) heart rate at different times of the study, showed a significant difference between different times. Heart rate decreased from recovery to 24 hours after surgery (p<0.001). As well, a highest significant difference was found between the ketamine group and group 3 (P=0.046) and the ketamine group and the control group (P=0.036) (figure 3)..

**Table 3:** Frequency distribution of analgesics intake in groups of study in patients under abdominal hysterectomy

**Groups**	**Pregabalin Group** **Person (%)**	**Ketamine Group** **Person (%)**	**Pregabalin-Ketamine** **Person** **(%)**	**Placebo** **Person (%)**
Step 1: Apotel (1 g)	10(28%)	12(34%)	10(28%)	14(40%)
Step 2: Morphine (2.5 mg)	6(17%)	13(37%)	9(25%)	17(48%)
Total	16	25	19	33

## Discussion

Patients undergoing major abdominal surgery, such as abdominal hysterectomy, usually experience severe pain during the early hours after surgery. Control of acute pain in these patients is very important. Different methods can be used to control postoperative pain. However, the use of any method for controlling pain during and after surgery has some disadvantages. For example, the use of opioids can cause respiratory suppression and nausea and vomiting. Likewise, the use of non-steroidal anti-inflammatory drugs can cause digestive complications(4, 19).

In our study, the changes in pain score measured with the VAS were studied over a period of 24 hours. Data analysis showed that in all groups, the pain at different times of the study was significantly different and had a decreasing trend over the course of 24 hours, which is expected to be due to the administration of analgesics. By comparing the pain at different times, it was shown that the mean pain score in the pregabalin group was lower than the other groups. More, the pain score in the times of study between the pregabalin and ketamine groups in the first 6 hours was significantly different, which could indicate better and faster effectiveness of pregabalin. However, there was no significant difference between these two groups in the 18th and 24th hours after surgery, but the mean pain score was lower in the pregabalin group. There was no significant difference in the pain scores of the pregabalin group and group pregabalin-ketamine group in any times of the study, although the mean pain score in the pregabalin group was lower in this section too. Baidya et al. reported that the analgesic effects of pregabalin were retained for up to 6 hours, and afterwards, due to decreased plasma concentrations of the drug, its analgesic effects decreased, but in this study, given that the pain was measured with the VAS at different times, this reduction was not distinguishable(20). Bhardwaj S, investigated the analgesic effects of oral pregabalin (150 mg) and the mix of oral pregabalin (150 mg) and ketamine (0.15 mg/kg) in the initial 24 hours after abdominal surgery. 

They reported that the mean score of VAS in the earliest hours after surgery was higher in the pregabalin group, but with the passage of time, the mean score of VAS in the group receiving the pregabalin was lower than that of the other group. Generally, there was no significant difference between the two groups during the time of the study. The results of our study and Bhardwaj’s study are generally the same, but the difference can be due to the difference in the dosage of administered medications(21). In the study of Mahran et al. which was carried out on 90 patients under breast cancer, patients were evaluated in three groups receiving placebo, 150 mg oral pregabalin, and 0.5 mg/kg intravenous ketamine for postoperative pain during 24 hours after surgery and the mean score of VAS (in rest and motion mode) was studied, of which there was no significant difference between the mean scores in the three groups. The interesting point in Mahran’s study is that, morphine was continuously infused with a PCA pump for all patients, which probably affected the pain level in all patients, indicated the effect of opioids on the pain control and morphine levels in the pregabalin group were lower than in the ketamine group as well as in the control group (6). Although, in our study, the amount of analgesia in the pregabalin group and group pregabalin-ketamine group was lower than that of the ketamine and control groups. Haliloglu et al., compared the antinociceptive effects of oral pregabalin (300 mg) and ketamine (0.3 mg/kg + 0.5 mg/kg/h infusion) with the control group in patients undergoing laparoscopic cholecystectomy. They showed that the amount of tramadol in the pregabalin group and ketamine group was significantly lower than the control group (22). Also, the need for additional diclofenac in the pregabalin group was lower than that of ketamine and control groups. In our study like the Haliloglu’s study, the amount of received additional analgesia in the pregabalin group was lower than other groups. Although in a study by Haliloglu et al., there was no significant difference between the need for an additional analgesia in the ketamine and pregabalin groups, in our study, more than 70% of patients in the ketamine group requested an additional analgesia. This difference may be due to the administration of ketamine infusion in addition to the initial dose in the Haliloglu et al.’s study and the administration of a single dose of ketamine in the present study. In our study, results were in agreement with a meta-analysis done in 2011. Zhang et al. showed that preoperative administration of pregabalin can reduce the first 24 h postoperative opioid consumption (23). 

In our study, the highest rate of postoperative nausea and vomiting was observed in the control group. This could be due to the use of extra pethidine in this group of patients. In the study of Haliloglu et al. there was no significant difference between groups in terms of incidence of nausea and vomiting. Scott and et al. reported that the administration of 150 mg pregabalin and 200 mg/kg celecoxib one hour before surgery in patients undergoing lumbar laminectomy reduced postoperative pain compared to the placebo. Also, the rate of nausea and vomiting in the pregabalin group was lower than the celecoxib group(24). In the study of Eskandar et al., they prescribed 300 mg of pregabalin to patients undergoing arthroscopic shoulder. They reported that the pregabalin reduces the postoperative pain and the amount of need for analgesia, and also the complications such as nausea and vomiting were not observed(25). Moreover, in study of Kim and et al., PONV is lesser in pregabalin group against placebo group (10).

In the present study, hemodynamic changes (heart rate and systolic blood pressure) were also evaluated. Overall, during the 24-hour evaluation, changes over time were significant, and patient hemodynamics were decreasing, and there was no significant decrease or increase in hemodynamic changes. The highest hemodynamic stability was observed in the pregabalin group and pregabalin-ketamine group. There was no significant difference between the different groups in terms of the mean of systolic blood pressure changes The findings of our study are in line with previous studies (26, 27). 

The most limitations of our study were the low number of patients enrolled in the study (for reasons such as coexisting disease, history of drug allergy, etc.) over a specified period of time. It is therefore suggested that similar studies be performed in a larger volume.

Our study shows oral administration of 300 mg pregabalin before surgery reduces postoperative pain and thus reduces the need foranalgesic drugs compared to the ketamine. Perhaps the reason is the difference in effect on the receptor P. It also reduces complications such as nausea and vomiting. Regarding the existence of questions about the effect of administration of different amounts of pregabalin and ketamine on postoperative pain, more studies are needed in this field to identify the antinociceptive effects of different doses and side effects of higher doses.
